# Seroprevalence of Hepatitis E Virus Varies Considerably Among Chinese Provinces

**DOI:** 10.5812/hepatmon.6194

**Published:** 2012-06-30

**Authors:** Chen Dong, Xing Dai, Jiuhong Liang, Min Dong, Jihong Meng

**Affiliations:** 1 School of Medicine, Southeast University, Nanjing, Jiangsu, China; 2 Zhongda Hospital, Southeast University, Nanjing, Jiangsu, China

**Keywords:** Hepatitis E, Seroprevalence, China

## Abstract

**Background:**

Hepatitis E is a common infection in China, but few studies have been carried out to compare regional and ethnic factors in its prevalence.

**Objectives:**

To characterize the seroprevalence of anti-HEV IgM and IgG in the general population of 11 Chinese provinces and in the people from different ethnic minorities.

**Materials and Methods:**

Sera from 14208 people including 723 people from four ethnic minorities were screened for anti-HEV IgM and IgG by enzyme-linked immunosorbent assay (ELISA). For the anti-HEV IgM positive samples, reverse transcription-polymerase chain reaction (RT-PCR) was carried out for the detection of HEV RNA.

**Results:**

The overall prevalence of anti-HEV IgG was 19.7%. The highest rate was 35.7% in Guizhou, while the lowest rate was 5.5% in Shanxi. Significantly higher rates were found among males compared to females in Hebei and Hunan province, and among females compared to males in Chongqing and Shannxi. In Guizhou, the prevalence rates among the Buyi, Miao, Shui and Han ethnic groups were 41.8%, 32.0%, 37.5% and 34.7%, respectively, which were not significantly different. The results also showed that the anti-HEV IgG detection rates increased with age for each ethnic group. Additionally, four samples were tested positive for anti-HEV IgM but HEV RNA was not detectable.

**Conclusions:**

HEV prevalence varies considerably among Chinese provinces. Thus, prevention and control programs including vaccination could be specifically targeted to people living in regions with relatively higher prevalences.

## 1. Background

Hepatitis E virus (HEV) is a single-strand, non-enveloped virus with an RNA genome of ~7.5 kb in length. It is the only member of the genus Hepevirus in the family Hepeviridae [1]. Hepatitis E, caused by HEV, is responsible for nearly 50% of cases of acute viral hepatitis in developing countries of Asia, Africa and Latin America where sanitary conditions are suboptimal [2][3][4]. Acute infection primarily affects young adults between 15 and 40 years of age and is generally mild, but the mortality rate is particularly high (10% - 40%) among pregnant women [5]. Chronic HEV infection, although rare, has been reported in immune suppressed people [6][7][8][9]. The epidemiology of HEV is complex [10][11][12]. Studies in endemic regions indicate high seroprevalence rates ranging from 15% to 60% [10]. In China, hepatitis E epidemics have largely been associated with consumption of focally contaminated water [13]. There is gathering evidence to suggest that HEV is also a zoonosis [14][15][16]. Although HEV infection with eating raw or inadequately cooked meat and offal from dear, boars and pigs has been reported [17][18][19], these have not been reported in China. Several studies have been carried out to estimate the seroprevalence of HEV in Chinese population [20][21][22][23][24][25].Relatively high seroprevalence rates were found in pig farmers, old people and people living in rural areas, which contrast with general population, children and people who lived in urban districts [21][22][24]. These studies were, however, carried out in populations confined to relatively small locales.

## 2. Objectives

The present study is an attempt to investigate the anti-HEV IgM and IgG in the general population of 11 Chinese provinces. Furthermore, for one province, where the highest prevalence rate was observed, we also included people belonging to minority ethnic groups.

## 3. Materials and Methods

### 3.1. Subjects

The study population consisted of 14208 apparently healthy people. These people, were community survey participants came from 11 provinces, include Hebei (n = 1374), Shanxi (n = 786), Beijing (n = 500), Jilin (n = 1177), Jiangsu (n = 1941), Anhui (n = 1529), Chongqing (n = 797), Hunan (n = 2181), Henan (n = 1866), Shannxi (n = 870) and Guizhou (n = 1187). In Guizhou, the following minority ethnic groups were studied: Miao (n = 225), Shui (n = 404), Buyi (n = 91) and Yi (n = 3); the remaining belongs to Han, which is the predominant ethnic group in China.

### 3.2. Ethical Considerations

This work has been carried out in accordance with the Declaration of Helsinki (2000) of the World Medical Association and was reviewed and approved by the Research Ethics Committee of Southeast University, China.

### 3.3. Serum Sampling

Serum samples were collected from June 2006 to June 2008 and stored at -70˚C until analyzed.

### 3.4. Detection of Antibodies Against HEV

The protocols used for detection of anti-HEV IgG and IgM were determined by an indirect (sandwich) enzyme immunoassay developed by Meng et al. with slightly modification [21]. Briefly, polystyrene plates were coated with antigens and stored at 4˚C until use. Test sera, with a 1:20 dilution, were added into the wells and incubated at 37˚C for 45 minutes. After washing, horseradish peroxidase-labeled goat-anti-human IgG or IgM was added to each well and then incubated at 37˚C for 45 minutes. The plates were washed five times with wash buffer after this incubation. After washing, TMB was added and incubated for 15 min. The reaction was stopped by adding H2SO4. The absorbance was read at 450 nm. The cutoff values for the assay of anti-HEV IgG and IgM were set at 0.252 and 0.386, respectively.

### 3.5. Detection of HEV RNA

For the anti-HEV IgM positive samples, reverse transcription-polymerase chain reaction (RT-PCR) was carried out for detection of HEV RNA. HEV RNA was purified from 100 µl serum, reverse transcribed, and then subjected to nested PCR as described previously [14]. Amplicons were separated by agarose gel electrophoresis with size markers and visualized by ethidium bromide fluorescence.

### 3.6. Statistical Methods

Chi-Square tests were used to compare the anti-HEV positive proportions between different groups. Statistical analysis was carried out by using SPSS version 15.0 for Windows.

## 4. Results

### 4.1. Prevalence of Anti-HEV IgG and IgM in Chinese General Population

In total, 14208 serum samples originating from people living in 11 provinces in China were collected. Serum samples were tested for anti-HEV IgG and IgM antibodies by using an indirect ELISA as described in materials and methods. The overall prevalence of anti-HEV IgG was 19.7%, 20.1% in males, and 19% in females, with no significant difference between sexes. For anti-HEV IgM detection, positivity was determined in four samples. Three male samples were from Jiangsu [2] and Hunan [1]. And the one female sample was from Anhui province. However, HEV RNA could not be isolated from these four samples by RT-PCR.

### 4.2. Seroprevalence of Anti-HEV IgG in Different Provinces

**Figure 1:**
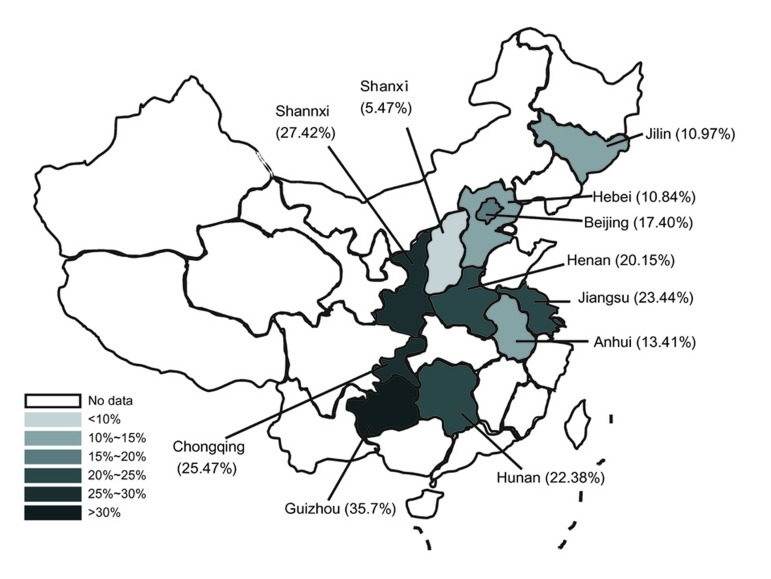
A Map Indicating Different Seroprevalence of HEV Among Provinces in China

As shown in Figure 1, the detection rates of anti-HEV IgG varied among the Chinese provinces. The highest prevalence was 35.7%, in Guizhou, and the lowest was 5.5%, in Shanxi province. The seroprevalence in Jilin, Hebei, Anhui, Beijing and Henan ranged from 10% to 20% while those in Hunan, Jiangsu, Chongqing and Shannxi ranged from 20% to 30%. Significantly higher prevalences in males compared to females were observed from Hebei (13% vs. 7.6%, respectively, P = 0.001) and Hunan province (24.3% vs. 19.6%, respectively, P = 0.009). By contrast, higher prevelances among women compared to men were observed in Chongqing (20.9% vs. 31.8%, respectively, P = 0.0001) and in Shannxi province (23.5% vs. 31.79%, respectively, P = 0.007). In the other seven provinces, there were no significant associations in prevalences between sexes (Table 1).

**Table 1:** Seroprevalence of Anti-HEV in 11 Provinces, China a

**Province**	**Male**			**Female**			**Total of Each Province**		
	**HEV IgG Positive, No. (%)**	**HEV IgG Negative, No.**	**Total, No.**	**HEV IgG Positive, No. (%)**	**HEV IgG Negative, No.**	**Total, No.**	**HEV IgG Positive, No. (%)**	**HEV IgG Negative, No.**	**Total, No.**
Jilin	74 (11)	600	674	53 (10.5)	450	503	127 (10.8)	1050	1177
Hebei	107 (13.1) b	713	820	42 (7.6)	512	554	149 (10.8)	1225	1374
Anhui	125 (14.2)	756	881	80 (12.4)	568	648	205 (13.4)	1324	1529
Beijing	47 (19)	200	247	40 (15.8)	213	253	87 (17.4)	413	500
Henan	212 (19.6)	870	1082	164 (20.9)	620	784	376 (20.2)	1490	1866
Hunan	310 (24.4) c	963	1273	178 (19.6)	730	908	488 (22.4)	1693	2181
Jiangsu	246 (24.7)	750	996	209 (22.1)	736	945	455 (23.4)	1486	1941
Chongqing	96 (20.9)^d^	364	460	107 (31.8)	230	337	203 (25.5)	594	797
Shannxi	113 (23.5) e	367	480	124 (31.8)	266	390	237 (27.2)	633	870
Guizhou	266 (37.6)	441	707	157 (32.7)	323	480	423 (35.7)	764	1187
Total	1614 (20.1)	6401	8015	1179 (19)	5014	6193	2793 (19.7)	11415	14208

^a^ Chi-Square Tests were used to compare the anti-HEV positive proportions between males and females

^b^ Male vs. Female: x(2)=10.223, P = 0.001

^c^ Male vs. Female: x(2)=6.880, P = 0.009

^d^ Male vs. Female: x(2)=12.132, P = 0.000

^e^ Male vs. Female: x(2)=7.359, P = 0.007

### 4.3. Seroprevalence of Anti-HEV IgG in the Minorities

In the present study, the samples were originating from Guizhou province included five different minorities. The seroprevalence of anti-HEV IgG among Chinese ethnic minorities has also been analyzed. As seen in Table 2, the overall prevalence was 41.8%, 32%, 37.5% and 34.7% in Buyi, Miao, Shui and Han ethnic groups, respectively, despite the fact, these differences were not significant. There was also no significant difference between men and women in four different ethnic groups. The anti-HEV IgG detection rates increased with age for each ethnic group. Although these age-wise differences were not statistically significant in the Buyi, Miao and Han ethnic groups, the difference in the Shui group was also significant.

**Table 2:** Seroprevalence of Anti-HEV in Minorities, China a

		**Male**			**Female**			**Total**		
**Ethnic Groups^b^**	**Age, y^c^**	**Anti-HEV Positive, No. (%)**	**Anti-HEV Negative, No.**	**Total, No.**	**Anti-HEV Positive, No. (%)**	**Anti-HEV Negative, No.**	**Total, No.**	**Anti-HEV Positive, No. (%)**	**Anti-HEV Negative, No.**	**Total, No.**
Buyi^d^	≤ 18	4 (40)	6	10	1 (20.0)	5	6	5 (31.2)	11	16
Buyi	18~60	15 (42.9)	20	35	11 (40.7)	16	27	26 (41.9)	36	62
Buyi	> 60	4 (50)	4	8	3 (60.0)	2	5	7 (53.8)	6	13
Buyi	Total	23 (43.4)	30	53	15 (39.5)	23	38	38 (41.8)	53	91
Miao^e^	< 18	9 (31.0)	20	29	4 (20.0)	16	20	13 (26.5)	36	49
Miao	18~60	29 (34.9)	54	83	22 (31.9)	47	69	51 (33.6)	101	152
Miao	> 60	4 (40.0)	6	10	4 (28.6)	10	14	8 (33.3)	16	24
Miao	Total	42 (34.4)	80	122	30 (29.1)	73	103	72 (32.0)	153	225
Shui^f^	< 18	19 (24.4)	59	78	8 (23.5)	26	34	27 (24.1)	85	112
Shui	18~60	61 (41.8)	85	146	37 (38.5)	59	96	98 (40.5)	144	242
Shui	> 60	20 (48.8)	21	41	6 (66.7)	3	9	26 (52.0)	24	50
Shui	Total	100 (37.7)	165	265	51 (36.7)	88	139	151 (37.5)	253	404
Han^g^	< 18	12 (26.7)	33	45	7 (25.0)	21	28	19 (26.0)	54	73
Han	18~60	68 (38.6)	108	176	42 (30.7)	95	137	110 (35.1)	203	313
Han	> 60	20 (44.4)	25	45	12 (36.4)	21	33	32 (41.0)	46	78
Han	Total	100 (37.6)	166	266	61 (30.8)	137	198	161 (34.7)	303	464

^a^ Chi-Square Tests were used to compare the anti-HEV positive proportions between males and females in different age groups.

^b^ No significant difference among four ethnic groups (x^2^ = 3.591, P > 0.05)

^c^ Chi-square test for trend was used to analyze the trend change of the anti-HEV positive proportions from group < 18 years to group 60 years old and above.

^d^ x^2^ = 1.489, P > 0.05

^e^ x^2^ = 3.702, P = 0.054

^f^ x^2^ = 0.586, P > 0.05

^g^ x^2^ = 13.741, P < 0.001.

## 5. Conclusions

We conducted a large population-based serosurvey to determine the prevalence of HEV infection in China. Geographically, the 11 provinces sampled in this study nearly occupy 0.33 portion of China population. The overall prevalence was 19.7%. Previous studies, conducted in eastern and northeastern China, revealed that the anti-HEV-positive rate was about 20% to 50 % [21][24]. This difference could have arisen because of differences in the study sample sizes or may reflect true regional differences. Our findings, representing 11 provinces, revealed markedly different rates among the provinces, ranging from 5.47% to 35.7%. This disparity suggested that the extent of exposure to HEV varied significantly among different provinces. Factors explaining these differences could include differences in living and sanitary conditions. We included a study of the sero epidemiology of HEV in Chinese ethnic minorities. Besides the Han group, Guizhou Province has more than 10 ethnic minorities. We observed that, among Miao, Shui and Buyi groups, the HEV seroprevalence rates were similar to Han in Guizhou province, which suggests that the four groups studied shared the same risk factors for HEV infection. The prevalence rate in Han group in Guizhou was significantly higher than the rates observed in other provinces studied, where the Han ethnic group is predominant. As Guizhou is relatively underdeveloped compared to other provinces, its low economic status, high crowding, abundant water resources and inadequate water treatment in some regions of this province may contribute to its higher endemicity. The data from the previous studies reported that HEV seropositivity rate varied considerably in northeast China [24][26]. In one paper, the overall prevalence of HEV was 47.7 % (143/300) [26]. This rate appears higher than 10.8% we observed in Jilin, which is also located in the northeast China. In another report from a study in Heilongjiang, Jilin and Liaoning provinces, the prevalence rates were between 4.1% to 30% in the general population [24]. The reason for the discrepancy between our data from Jilin and those from these 2 studies may be due to differences in the sensitivity and specificity of anti-HEV IgG assays employed [27][28]. Furthermore, the contribution of sampling errors cannot be excluded.

In summary, the overall anti-HEV seroprevalence rate among 11 Chinese provinces was 19.7%. The rates varied significantly in general population of different provinces in China, and in Guizhou, where the prevalence was highest, the rates were not significantly different among four ethnic groups sampled. Thus, prevention and control programs including vaccination could be specifically targeted to people living in regions with relativity higher prevalences.
